# Dr. Robert Newman

**Published:** 1903-11

**Authors:** 


### OBITUARY.

---

Dr. Robert Newman, of New York, died August 28, 1903, aged 73 years. Dr. Newman was a widely known advocate of electrolysis in the treatment of stricture of the urethra. He was also a frequent contributor to the medical journals and was a member of various local, state and national medical societies. He graduated at the Long Island College Hospital in 1863.

